# Draft genomes of the bile duct microbiome strains *Klebsiella pneumoniae* and *Enterococcus lactis* isolated from bilioenteric drainages with biofilm-forming abilities

**DOI:** 10.1128/mra.00202-24

**Published:** 2024-10-29

**Authors:** Güel Dumann, Oliver Rohland, Mostafa Y. Abdel-Glil, Rosalind J. Allen, Michael Bauer, Anne Busch

**Affiliations:** 1 Theoretical Microbial Ecology, Friedrich Schiller University, Jena, Germany; 2 Department of General, Visceral and Vascular Surgery, Jena University Hospital, Jena, Germany; 3 Friedrich-Loeffler-Institute, Institute of Bacterial Infections and Zoonoses (IBIZ), Jena, Germany; 4 Cluster of Excellence Balance of the Microverse, Friedrich Schiller University Jena, Jena, Germany; 5 Department of Anaesthesiology and Intensive Care Medicine, University Hospital Jena, Jena, Germany; Loyola University Chicago, Chicago, Illinois, USA

**Keywords:** bile duct, *Enterococcus*, *Klebsiella*, biofilms, antibiotic resistance, genome analysis, human microbiome

## Abstract

We describe the genetic properties of two strains isolated from the elusive bile duct microbiome from solid organ transplant patients. Bacterial strains *Enterococcus lactis* (MS-STENT-08-E-001) and *Klebsiella pneumoniae* (MS-STENT-01-M-001) were isolated from the biofilms of bile duct catheters.

## ANNOUNCEMENT

Liver transplant patients are at elevated risk of bacterial infections, particularly in the liver, where gut microbiome bacteria may infiltrate bile ducts. This can contribute to acute cholangitis (1–4). A better understanding of bacterial biofilm formation in bile duct catheters is needed, as Enterococci and Enterobacteriaceae commonly cause cholangitis (5). In 2023, MS-STENT-08-E-001 and MS-STENT-01-M-001 were isolated from bile duct catheters by ultrasonication of the biliary tip in 1 mL phospate-buffered saline for 10 seconds.

For sequencing, MS-STENT-08-E-001 was cultured on Enterococcus Selective Agar (NutriSelect, Darmstadt, Germany), and MS-STENT-01-M-001 was cultured on MacConkey Agar (Carl Roth, Karlsruhe, Germany), both at 37°C under aerobic conditions. Strain identification was additionally done via 16S gene sequencing [with primer 27F 5′-AGAGTTTGATCMTGGCTCAG-3′ and primer 1492R 5′-TACGGYTACCTTGTTACGACTT-3′ as described before (6)]; BLAST (vBLAST+ 2.3.0) (7, 8) and PubMLST (12/2023) (9, 10) identified MS-STENT-08-E-001 as *Enterococcus lactis* and MS-STENT-01-M-001 as *Klebsiella pneumoniae* (8, 9, 11). Standard parameters were used for all software in this paper unless specified otherwise. The DNA library was prepared using the Nextera DNA Flex microbial colony extraction protocol and Illumina’s standard protocol for Illumina DNA Preparation (12, 13). Paired-end sequencing was performed on the iSeq platform with a 300-cycle iSeq reagent kit resulting in MS-STENT-08-E-001 in 1,818,202 reads and MS-STENT-08-E-001 in 2,874,770 reads (13). Reads were quality-controlled and trimmed using BBTrim (https://jgi.doe.gov/data-and-tools/software-tools/bbtools/bb-tools-user-guide/) following FastQC analysis, within Geneious Prime (v. 2023.2) (14). *De novo* assembly was performed using SPAdes (v. 3.15.4) with default parameters, and all contigs shorter than 500 bp were filtered out (15).

For the *Enterococcus lactis* isolate, the genome contained 62 contigs, an N50 of 114,878 bp, and a mean sequencing depth of 140×. The genomic sequence was 2,744,144 bp in length, with a G+C content of 38.2%. Genome annotation by the Prokaryotic Genome Annotation Pipeline (PGAP, v6.6) identified 2,719 genes (16). ResFinder (v4.4.1) revealed the presence of two acquired antimicrobial resistance genes: *aac(6')-Ii* and *msr(C)* (13). The predicted antibiotic resistances are gentamicin, tobramycin, dibekacin, netilmicin, sisomicin, erythromycin, telithromycin, quinupristin, pristinamycin 1a, and virginiamycin s1 (13).

The genome of the *Klebsiella pneumoniae* isolate comprised 47 contigs, with an N50 contig of 230,642 bp with at least coverage of 40×. It spanned a length of 5,477,030 bp with a G+C content of 57.3%. PGAP (v6.6) annotation identified 5,297 genes (16). Using Geneious Prime, the genome assembly was mapped against a collection of biofilm-associated genes (17), revealing the presence of *fimH, bcsABZC_AP010953,* and *crl*. ResFinder (v4.4.1) detected four acquired antimicrobial resistance genes: *blaSHV-99*, *fosA6*, *OqxB*, and *OqxA* (13). The predicted antibiotic resistances included ciprofloxacin, nalidixic acid, amoxicillin, ampicillin, cefepime, cefotaxime, ceftazidime, piperacillin, aztreonam, ticarcillin, ceftriaxone, trimethoprim, fosfomycin, and chloramphenicol (13).

A Micronaut plate system was utilized to assess *in vivo* resistance predictions using brain heart infusion media. The Micronaut AST system uses standardized microdilution procedures to determine minimal inhibitory concentrations, and EUCAST guidelines can be adapted (18). This suggested a prediction bias in the ResFinder (v4.4.1) for MS-STENT-08-E-001 (false negative), eventually attributed to biases within the database or due to the different medium (Table 1).

**TABLE 1:** *In vivo* testing and *in silico* antibiotic resistance prediction

*Enterococcus lactis* (MS-STENT-08-E-001)				*Klebsiella pneumoniae* (MS-STENT-01-M-001)			
Antibiotic tested	*In vivo* (MIC values)	EUCAST	***In silico***	Antibiotic tested	*In vivo* (MIC values)	EUCAST	***In silico***
Ampicillin/sulbactam	<1 µg/mL	Resistant ≤8 µg/mL	Sensitive	Ampicillin/sulbactam	<1 µg/mL	Resistant ≤8 µg/mL	Resistant
Cefotaxime	≤2 µg/mL	Not applicable	Sensitive	Cefotaxime	<0.5 µg/mL, s	Resistant ≤2 µg/mL	Resistant
Cefpodoxime proxetil	≤4 µg/mL	Not applicable	Not applicable	Cefpodoxime proxetil	<0.25 µg/mL	Resistant ≤1 µg/mL	Not applicable
Ciprofloxacin	≤0.5 µg/ml	Resistant ≤4 µg/mL	Sensitive	Ciprofloxacin	<0.125 µg/mL, s	Resistant ≤0.5 µg/mL	Resistant
Fosfomycin	≤32 µg/mL	Not applicable	Sensitive	Fosfomycin	≤64 µg/mL	Not applicable	Resistant
Linezolid	<4 µg/mL	Resistant ≤4 µg/mL	Sensitive	Linezolid	≤8 µg/mL	Not applicable	Sensitive
Mecillinam	≤16 µg/mL	Not applicable	Not applicable	Mecillinam	≤8 µg/mL	Resistant ≤8 µg/mL	Not applicable
Nitrofurantoin	≤32 µg/mL	Resistant ≤64 µg/mL	Not applicable	Nitrofurantoin	≤32 µg/mL	Resistant ≤64 µg/mL	Not applicable
Piperacillin/tazobactam	≤0.5 µg/mL	Not applicable	Sensitive	Piperacillin/tazobactam	≤0.25 µg/mL	Resistant ≤8 µg/mL	Resistant
Trimethoprim/sulfamethoxazole	<0.05 µg/mL	Not applicable	Sensitive	Trimethoprim/sulfamethoxazole	<0.05 µg/mL	Resistant ≤4 µg/mL	Resistant

## Data Availability

The raw reads and the assembled genomes for the *Klebsiella pneumoniae* isolate are available for this project under reference PRNJA 1041718, Biosample ID SAMN38290122 with the assembly GCA_033949525.1. The raw reads and the assembled genomes for the *Enterococcus lactis* isolate are available in the BioProject PRNJA1041721, Biosample ID SAMN38290137 with the assembly GCA_033949555.1.
